# (*E*)-3-Methyl-5-(4-methyl­phen­oxy)-1-phenyl-1*H*-pyrazole-4-carbaldehyde *O*-[(2-chloro-1,3-thia­zol-5-yl)meth­yl]oxime

**DOI:** 10.1107/S1600536811006702

**Published:** 2011-02-26

**Authors:** Hong Dai, Yu-Ting Zhang, Yu-Jun Shi, Wen-Wen Zhang, Yong-Jun Shen

**Affiliations:** a College of Chemistry and Chemical Engineering, Nantong University, Nantong 226019, People’s Republic of China

## Abstract

In the title compound, C_22_H_19_ClN_4_O_2_S, the planes of the benzene ring, the substituted phenyl ring and the thia­zole ring make dihedral angles of 18.4 (3), 88.9 (2) and 63.0 (3)°, respectively, with the pyrazole ring.

## Related literature

For the biological activity of pyrazole oxime ether derivatives, see: Drabek (1992 ▶); Motoba *et al.* (2000 ▶); Park *et al.* (2005 ▶); Watanabe *et al.* (2001 ▶). For the bioactivity of compounds containing a thia­zole ring, see: Araki (2004 ▶); Fahmy & Bekhit (2002 ▶); Manabe *et al.* (2003 ▶); Zhang *et al.* (2000 ▶).
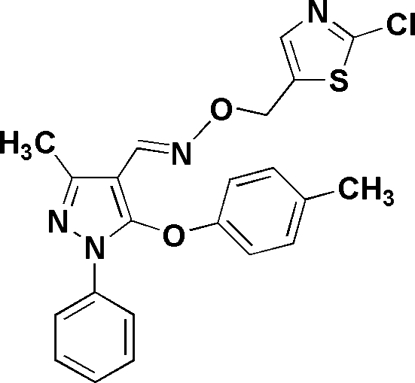


## Experimental

### 

#### Crystal data

C_22_H_19_ClN_4_O_2_S
*M* *_r_* = 438.92
Triclinic, 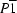

*a* = 8.114 (3) Å
*b* = 11.452 (4) Å
*c* = 12.494 (4) Å
α = 102.700 (6)°
β = 107.885 (6)°
γ = 93.634 (7)°
*V* = 1067.1 (6) Å^3^
*Z* = 2
Mo *K*α radiation
μ = 0.30 mm^−1^
*T* = 294 K
0.20 × 0.18 × 0.10 mm

#### Data collection

Bruker SMART CCD area-detector diffractometer
Absorption correction: multi-scan (*SADABS*; Sheldrick, 1996 ▶) *T*_min_ = 0.938, *T*_max_ = 0.968
5562 measured reflections
3755 independent reflections
2030 reflections with *I* > 2σ(*I*)
*R*_int_ = 0.029

#### Refinement

*R*[*F* ^2^ > 2σ(*F* ^2^)] = 0.049
*wR*(*F* ^2^) = 0.124
*S* = 1.00
3755 reflections
273 parameters
H-atom parameters constrained
Δρ_max_ = 0.17 e Å^−3^
Δρ_min_ = −0.19 e Å^−3^

### 

Data collection: *SMART* (Bruker, 2000 ▶); cell refinement: *SAINT* (Bruker, 2000 ▶); data reduction: *SAINT*; program(s) used to solve structure: *SHELXS97* (Sheldrick, 2008 ▶); program(s) used to refine structure: *SHELXL97* (Sheldrick, 2008 ▶); molecular graphics: *SHELXTL* (Sheldrick, 2008 ▶); software used to prepare material for publication: *SHELXTL*.

## Supplementary Material

Crystal structure: contains datablocks global, I. DOI: 10.1107/S1600536811006702/ds2094sup1.cif

Structure factors: contains datablocks I. DOI: 10.1107/S1600536811006702/ds2094Isup2.hkl

Additional supplementary materials:  crystallographic information; 3D view; checkCIF report

## Figures and Tables

**Fig. 1.**
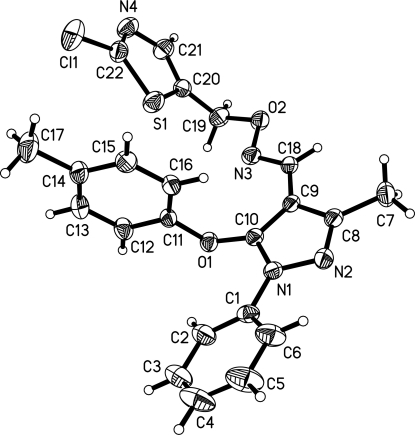
View of the title compound, with displacement ellipsoids drawn at the 30% probability level.

## supplementary crystallographic information

### Comment

In the past few years, pyrazole oxime ethers have been found to exhibit a wide range of bioactivities, such as fungicidal, insecticidal, acaricidal and anticancer activities (Drabek, 1992; Motoba *et al.*, 2000; Watanabe *et al.*, 2001;Park *et al.*, 2005). In addition, the biological activity of thiazole derivatives has been the subject of intense interest for past decades. They are widely used as fungicide, insecticide, herbicide and antitumor agents (Zhang *et al.*, 2000; Fahmy & Bekhit, 2002; Manabe *et al.*, 2003; Araki,2004). Having the above facts in mind and in continuation of our efforts to explore more biologically active moleculars, we synthesized a series of pyrazole oxime ether compounds containing a thiazole moiety. Herein we report the crystal structure of the title compound. The molecule of the title compound (Fig.1)contains four planar rings, the benzene ring (p1: C1/C2/C3/C4/C5/C6), the substituted phenyl ring (p2: C11/C12/C13/C14/C15/C16),the thiazole ring(p3: C20/C21/N4/C22/S1) and the pyrazole ring(p4: N1/N2/C8/C9/C10).The planes of p1, p2 and p3 make dihedral angles of 18.4 (3)°, 88.9 (2) ° and 63.0 (3) °,respectively, with p4.

### Experimental

To a well stirred solution of 1-phenyl-3-methyl-5-(4-methylphenoxy)-1*H*- pyrazole-4-carbaldehyde oxime (3 mmol),and powdered potassium carbonate (6 mmol) in 20 ml of anhydrous acetone, was added 2-chloro-5-chloromethyl thiazole (3.3 mmol) at room temperature. The mixture was heated to reflux for 10 h. The solvent was evaporated under reduced pressure, and then 80 ml of dichloromethane was added to the residue. The organic layer was washed with saturated brine(3 * 20 ml),and dried over anhydrous magnesium sulfate. After removal of the solvent, the residue was separated by column chromatography on silica gel with petroleum ether/ethyl acetate(6:1 *v*/*v*) as eluent, and recrystallized from ethyl acetate to give a colourless crystal.

### Refinement

All H atoms were placed in calculated positions, with C–H = 0.93, 0.96 and 0.97 Å, and included in the final cycles of refinement using a riding model, with *U*_iso_(H) = 1.2*U*_eq_(C).

### Crystal data

**Table:**

C_22_H_19_ClN_4_O_2_S	*Z* = 2
*M**_r_* = 438.92	*F*(000) = 456
Triclinic, *P*1	*D*_x_ = 1.366 Mg m^−^^3^
Hall symbol: -P 1	Mo *K*α radiation, λ = 0.71073 Å
*a* = 8.114 (3) Å	Cell parameters from 1218 reflections
*b* = 11.452 (4) Å	θ = 2.7–22.3°
*c* = 12.494 (4) Å	µ = 0.30 mm^−^^1^
α = 102.700 (6)°	*T* = 294 K
β = 107.885 (6)°	Triclinic, colourless
γ = 93.634 (7)°	0.20 × 0.18 × 0.10 mm
*V* = 1067.1 (6) Å^3^	

### Data collection

**Table:**

Bruker SMART CCD area-detector diffractometer	3755 independent reflections
Radiation source: fine-focus sealed tube	2030 reflections with *I* > 2σ(*I*)
graphite	*R*_int_ = 0.029
φ and ω scans	θ_max_ = 25.0°, θ_min_ = 1.8°
Absorption correction: multi-scan (*SADABS*; Sheldrick, 1996)	*h* = −9→9
*T*_min_ = 0.938, *T*_max_ = 0.968	*k* = −10→13
5562 measured reflections	*l* = −14→14

### Refinement

**Table:**

Refinement on *F*^2^	Primary atom site location: structure-invariant direct methods
Least-squares matrix: full	Secondary atom site location: difference Fourier map
*R*[*F*^2^ > 2σ(*F*^2^)] = 0.049	Hydrogen site location: inferred from neighbouring sites
*wR*(*F*^2^) = 0.124	H-atom parameters constrained
*S* = 1.00	*w* = 1/[σ^2^(*F*_o_^2^) + (0.049*P*)^2^ + 0.1132*P*] where *P* = (*F*_o_^2^ + 2*F*_c_^2^)/3
3755 reflections	(Δ/σ)_max_ = 0.001
273 parameters	Δρ_max_ = 0.17 e Å^−^^3^
0 restraints	Δρ_min_ = −0.19 e Å^−^^3^

### Special details

**Table:**

Geometry. All e.s.d.'s (except the e.s.d. in the dihedral angle between two l.s. planes) are estimated using the full covariance matrix. The cell e.s.d.'s are taken into account individually in the estimation of e.s.d.'s in distances, angles and torsion angles; correlations between e.s.d.'s in cell parameters are only used when they are defined by crystal symmetry. An approximate (isotropic) treatment of cell e.s.d.'s is used for estimating e.s.d.'s involving l.s. planes.
Refinement. Refinement of *F*^2^ against ALL reflections. The weighted *R*-factor *wR* and goodness of fit *S* are based on *F*^2^, conventional *R*-factors *R* are based on *F*, with *F* set to zero for negative *F*^2^. The threshold expression of *F*^2^ > σ(*F*^2^) is used only for calculating *R*-factors(gt) *etc*. and is not relevant to the choice of reflections for refinement. *R*-factors based on *F*^2^ are statistically about twice as large as those based on *F*, and *R*- factors based on ALL data will be even larger.

### Fractional atomic coordinates and isotropic or equivalent isotropic displacement parameters (Å^2^)

**Table:**

	*x*	*y*	*z*	*U*_iso_*/*U*_eq_	
S1	0.45270 (11)	0.85178 (9)	0.33662 (8)	0.0647 (3)	
Cl1	0.61728 (16)	0.68418 (11)	0.47383 (9)	0.0975 (4)	
O1	−0.0621 (2)	0.84964 (18)	0.10876 (17)	0.0450 (5)	
O2	0.4577 (3)	1.0355 (2)	0.16837 (17)	0.0530 (6)	
N1	−0.2014 (3)	0.7626 (2)	−0.0923 (2)	0.0434 (7)	
N2	−0.1696 (3)	0.7554 (2)	−0.1953 (2)	0.0510 (7)	
N3	0.2917 (3)	0.9627 (2)	0.1279 (2)	0.0479 (7)	
N4	0.7640 (4)	0.8986 (3)	0.4813 (2)	0.0660 (9)	
C1	−0.3633 (4)	0.7038 (3)	−0.0939 (3)	0.0471 (8)	
C2	−0.4206 (4)	0.7326 (3)	0.0001 (3)	0.0636 (10)	
H2	−0.3572	0.7937	0.0653	0.076*	
C3	−0.5742 (5)	0.6692 (4)	−0.0040 (4)	0.0768 (12)	
H3	−0.6126	0.6870	0.0599	0.092*	
C4	−0.6702 (5)	0.5811 (4)	−0.0999 (5)	0.0914 (14)	
H4	−0.7723	0.5384	−0.1009	0.110*	
C5	−0.6157 (5)	0.5561 (4)	−0.1939 (4)	0.0941 (14)	
H5	−0.6828	0.4975	−0.2602	0.113*	
C6	−0.4610 (5)	0.6171 (4)	−0.1921 (3)	0.0719 (11)	
H6	−0.4240	0.5994	−0.2566	0.086*	
C7	0.0690 (4)	0.8267 (4)	−0.2568 (3)	0.0676 (11)	
H7A	0.1699	0.7855	−0.2474	0.101*	
H7B	0.1031	0.9108	−0.2490	0.101*	
H7C	−0.0152	0.7919	−0.3324	0.101*	
C8	−0.0104 (4)	0.8142 (3)	−0.1657 (3)	0.0465 (8)	
C9	0.0656 (4)	0.8599 (3)	−0.0440 (2)	0.0400 (8)	
C10	−0.0610 (4)	0.8238 (3)	−0.0019 (3)	0.0395 (8)	
C11	−0.0066 (4)	0.7658 (3)	0.1735 (2)	0.0408 (8)	
C12	−0.0280 (4)	0.7901 (3)	0.2804 (3)	0.0541 (9)	
H12	−0.0762	0.8582	0.3055	0.065*	
C13	0.0232 (4)	0.7116 (3)	0.3504 (3)	0.0591 (10)	
H13	0.0085	0.7278	0.4230	0.071*	
C14	0.0945 (4)	0.6111 (3)	0.3161 (3)	0.0531 (9)	
C15	0.1125 (4)	0.5889 (3)	0.2072 (3)	0.0550 (9)	
H15	0.1606	0.5209	0.1819	0.066*	
C16	0.0612 (4)	0.6647 (3)	0.1352 (3)	0.0449 (8)	
H16	0.0725	0.6474	0.0617	0.054*	
C17	0.1474 (5)	0.5256 (4)	0.3933 (3)	0.0871 (13)	
H17A	0.2202	0.5707	0.4695	0.131*	
H17B	0.2110	0.4678	0.3609	0.131*	
H17C	0.0447	0.4841	0.3984	0.131*	
C18	0.2345 (4)	0.9303 (3)	0.0182 (3)	0.0437 (8)	
H18	0.3035	0.9525	−0.0233	0.052*	
C19	0.5031 (4)	1.0792 (3)	0.2911 (3)	0.0565 (9)	
H19A	0.4004	1.1029	0.3096	0.068*	
H19B	0.5904	1.1505	0.3175	0.068*	
C20	0.5727 (4)	0.9879 (3)	0.3541 (3)	0.0468 (8)	
C21	0.7309 (4)	0.9944 (4)	0.4334 (3)	0.0606 (10)	
H21	0.8149	1.0620	0.4548	0.073*	
C22	0.6282 (5)	0.8196 (4)	0.4373 (3)	0.0581 (10)	

### Atomic displacement parameters (Å^2^)

**Table:**

	*U*^11^	*U*^22^	*U*^33^	*U*^12^	*U*^13^	*U*^23^
S1	0.0518 (6)	0.0703 (7)	0.0665 (6)	−0.0003 (5)	0.0059 (5)	0.0280 (5)
Cl1	0.1287 (10)	0.0848 (9)	0.0856 (8)	0.0232 (7)	0.0255 (7)	0.0467 (7)
O1	0.0519 (13)	0.0432 (14)	0.0441 (13)	0.0105 (11)	0.0182 (11)	0.0148 (11)
O2	0.0445 (13)	0.0612 (16)	0.0491 (14)	−0.0041 (11)	0.0079 (11)	0.0191 (12)
N1	0.0359 (15)	0.0482 (18)	0.0457 (16)	0.0056 (13)	0.0095 (13)	0.0168 (14)
N2	0.0474 (17)	0.062 (2)	0.0428 (16)	0.0036 (15)	0.0121 (14)	0.0170 (14)
N3	0.0375 (15)	0.0513 (18)	0.0510 (18)	−0.0010 (13)	0.0074 (13)	0.0170 (14)
N4	0.062 (2)	0.077 (2)	0.0519 (19)	0.0171 (19)	0.0049 (16)	0.0184 (18)
C1	0.0368 (18)	0.041 (2)	0.065 (2)	0.0078 (16)	0.0136 (18)	0.0205 (18)
C2	0.049 (2)	0.073 (3)	0.073 (3)	0.005 (2)	0.026 (2)	0.019 (2)
C3	0.059 (3)	0.084 (3)	0.099 (3)	0.009 (2)	0.042 (2)	0.025 (3)
C4	0.058 (3)	0.072 (3)	0.150 (5)	−0.003 (2)	0.051 (3)	0.020 (3)
C5	0.057 (3)	0.080 (3)	0.118 (4)	−0.016 (2)	0.025 (3)	−0.017 (3)
C6	0.049 (2)	0.070 (3)	0.084 (3)	−0.002 (2)	0.022 (2)	−0.002 (2)
C7	0.069 (2)	0.089 (3)	0.050 (2)	−0.001 (2)	0.0227 (19)	0.027 (2)
C8	0.0408 (19)	0.053 (2)	0.048 (2)	0.0056 (17)	0.0118 (16)	0.0210 (17)
C9	0.0383 (18)	0.043 (2)	0.0402 (19)	0.0085 (15)	0.0097 (15)	0.0171 (16)
C10	0.0390 (18)	0.039 (2)	0.0418 (19)	0.0092 (15)	0.0118 (16)	0.0146 (16)
C11	0.0399 (17)	0.045 (2)	0.0403 (19)	0.0021 (16)	0.0149 (15)	0.0149 (16)
C12	0.067 (2)	0.053 (2)	0.050 (2)	0.0139 (19)	0.0300 (18)	0.0120 (18)
C13	0.072 (2)	0.064 (3)	0.047 (2)	0.004 (2)	0.0266 (19)	0.016 (2)
C14	0.060 (2)	0.049 (2)	0.058 (2)	0.0042 (19)	0.0227 (19)	0.0241 (19)
C15	0.059 (2)	0.049 (2)	0.064 (2)	0.0136 (18)	0.0257 (18)	0.0194 (19)
C16	0.0498 (19)	0.044 (2)	0.0461 (19)	0.0081 (17)	0.0217 (16)	0.0128 (17)
C17	0.116 (3)	0.080 (3)	0.084 (3)	0.021 (3)	0.038 (3)	0.050 (3)
C18	0.0398 (18)	0.050 (2)	0.049 (2)	0.0083 (16)	0.0161 (17)	0.0251 (17)
C19	0.053 (2)	0.057 (2)	0.050 (2)	0.0042 (18)	0.0068 (17)	0.0090 (19)
C20	0.0438 (19)	0.054 (2)	0.0397 (18)	0.0060 (17)	0.0121 (16)	0.0077 (17)
C21	0.054 (2)	0.065 (3)	0.051 (2)	0.0035 (19)	0.0053 (18)	0.011 (2)
C22	0.069 (2)	0.064 (3)	0.047 (2)	0.018 (2)	0.0207 (19)	0.020 (2)

### Geometric parameters (Å, °)

**Table:**

S1—C22	1.708 (4)	C7—H7A	0.9600
S1—C20	1.718 (3)	C7—H7B	0.9600
Cl1—C22	1.714 (4)	C7—H7C	0.9600
O1—C10	1.352 (3)	C8—C9	1.414 (4)
O1—C11	1.397 (3)	C9—C10	1.370 (4)
O2—N3	1.421 (3)	C9—C18	1.436 (4)
O2—C19	1.424 (3)	C11—C16	1.368 (4)
N1—C10	1.351 (3)	C11—C12	1.370 (4)
N1—N2	1.375 (3)	C12—C13	1.384 (4)
N1—C1	1.430 (4)	C12—H12	0.9300
N2—C8	1.321 (4)	C13—C14	1.365 (5)
N3—C18	1.264 (4)	C13—H13	0.9300
N4—C22	1.272 (4)	C14—C15	1.383 (4)
N4—C21	1.365 (4)	C14—C17	1.512 (4)
C1—C2	1.374 (4)	C15—C16	1.377 (4)
C1—C6	1.374 (5)	C15—H15	0.9300
C2—C3	1.383 (5)	C16—H16	0.9300
C2—H2	0.9300	C17—H17A	0.9600
C3—C4	1.362 (6)	C17—H17B	0.9600
C3—H3	0.9300	C17—H17C	0.9600
C4—C5	1.358 (6)	C18—H18	0.9300
C4—H4	0.9300	C19—C20	1.482 (4)
C5—C6	1.388 (5)	C19—H19A	0.9700
C5—H5	0.9300	C19—H19B	0.9700
C6—H6	0.9300	C20—C21	1.345 (4)
C7—C8	1.497 (4)	C21—H21	0.9300
			
C22—S1—C20	88.36 (18)	C16—C11—C12	120.9 (3)
C10—O1—C11	117.5 (2)	C16—C11—O1	124.2 (3)
N3—O2—C19	107.2 (2)	C12—C11—O1	114.9 (3)
C10—N1—N2	110.3 (2)	C11—C12—C13	118.9 (3)
C10—N1—C1	130.4 (3)	C11—C12—H12	120.6
N2—N1—C1	119.3 (3)	C13—C12—H12	120.6
C8—N2—N1	105.2 (2)	C14—C13—C12	121.9 (3)
C18—N3—O2	110.9 (2)	C14—C13—H13	119.0
C22—N4—C21	108.2 (3)	C12—C13—H13	119.0
C2—C1—C6	120.4 (3)	C13—C14—C15	117.6 (3)
C2—C1—N1	121.3 (3)	C13—C14—C17	121.1 (3)
C6—C1—N1	118.3 (3)	C15—C14—C17	121.3 (3)
C1—C2—C3	118.9 (4)	C16—C15—C14	121.8 (3)
C1—C2—H2	120.6	C16—C15—H15	119.1
C3—C2—H2	120.6	C14—C15—H15	119.1
C4—C3—C2	121.2 (4)	C11—C16—C15	118.9 (3)
C4—C3—H3	119.4	C11—C16—H16	120.5
C2—C3—H3	119.4	C15—C16—H16	120.5
C5—C4—C3	119.5 (4)	C14—C17—H17A	109.5
C5—C4—H4	120.3	C14—C17—H17B	109.5
C3—C4—H4	120.3	H17A—C17—H17B	109.5
C4—C5—C6	120.7 (4)	C14—C17—H17C	109.5
C4—C5—H5	119.6	H17A—C17—H17C	109.5
C6—C5—H5	119.6	H17B—C17—H17C	109.5
C1—C6—C5	119.2 (4)	N3—C18—C9	121.6 (3)
C1—C6—H6	120.4	N3—C18—H18	119.2
C5—C6—H6	120.4	C9—C18—H18	119.2
C8—C7—H7A	109.5	O2—C19—C20	112.5 (3)
C8—C7—H7B	109.5	O2—C19—H19A	109.1
H7A—C7—H7B	109.5	C20—C19—H19A	109.1
C8—C7—H7C	109.5	O2—C19—H19B	109.1
H7A—C7—H7C	109.5	C20—C19—H19B	109.1
H7B—C7—H7C	109.5	H19A—C19—H19B	107.8
N2—C8—C9	112.1 (3)	C21—C20—C19	128.5 (3)
N2—C8—C7	120.5 (3)	C21—C20—S1	108.3 (3)
C9—C8—C7	127.4 (3)	C19—C20—S1	123.1 (2)
C10—C9—C8	103.7 (3)	C20—C21—N4	117.8 (3)
C10—C9—C18	129.1 (3)	C20—C21—H21	121.1
C8—C9—C18	127.2 (3)	N4—C21—H21	121.1
N1—C10—O1	122.1 (3)	N4—C22—S1	117.4 (3)
N1—C10—C9	108.8 (3)	N4—C22—Cl1	122.8 (3)
O1—C10—C9	128.9 (3)	S1—C22—Cl1	119.9 (2)
			
C10—N1—N2—C8	−0.8 (3)	C8—C9—C10—O1	−175.4 (3)
C1—N1—N2—C8	−178.1 (2)	C18—C9—C10—O1	3.0 (5)
C19—O2—N3—C18	173.5 (3)	C10—O1—C11—C16	5.4 (4)
C10—N1—C1—C2	20.1 (5)	C10—O1—C11—C12	−173.3 (3)
N2—N1—C1—C2	−163.2 (3)	C16—C11—C12—C13	1.2 (5)
C10—N1—C1—C6	−159.8 (3)	O1—C11—C12—C13	179.9 (3)
N2—N1—C1—C6	16.9 (4)	C11—C12—C13—C14	0.1 (5)
C6—C1—C2—C3	2.8 (5)	C12—C13—C14—C15	−0.8 (5)
N1—C1—C2—C3	−177.1 (3)	C12—C13—C14—C17	−179.2 (3)
C1—C2—C3—C4	−1.3 (6)	C13—C14—C15—C16	0.1 (5)
C2—C3—C4—C5	−1.0 (7)	C17—C14—C15—C16	178.6 (3)
C3—C4—C5—C6	1.8 (7)	C12—C11—C16—C15	−1.8 (4)
C2—C1—C6—C5	−2.0 (5)	O1—C11—C16—C15	179.6 (3)
N1—C1—C6—C5	177.9 (3)	C14—C15—C16—C11	1.1 (5)
C4—C5—C6—C1	−0.3 (6)	O2—N3—C18—C9	−177.5 (2)
N1—N2—C8—C9	0.4 (3)	C10—C9—C18—N3	4.6 (5)
N1—N2—C8—C7	−179.3 (3)	C8—C9—C18—N3	−177.4 (3)
N2—C8—C9—C10	0.0 (3)	N3—O2—C19—C20	79.6 (3)
C7—C8—C9—C10	179.7 (3)	O2—C19—C20—C21	119.4 (3)
N2—C8—C9—C18	−178.4 (3)	O2—C19—C20—S1	−62.1 (3)
C7—C8—C9—C18	1.3 (5)	C22—S1—C20—C21	0.0 (2)
N2—N1—C10—O1	176.1 (3)	C22—S1—C20—C19	−178.9 (3)
C1—N1—C10—O1	−7.0 (5)	C19—C20—C21—N4	178.9 (3)
N2—N1—C10—C9	0.8 (3)	S1—C20—C21—N4	0.1 (4)
C1—N1—C10—C9	177.8 (3)	C22—N4—C21—C20	−0.2 (4)
C11—O1—C10—N1	90.5 (3)	C21—N4—C22—S1	0.1 (4)
C11—O1—C10—C9	−95.2 (4)	C21—N4—C22—Cl1	179.3 (2)
C8—C9—C10—N1	−0.5 (3)	C20—S1—C22—N4	−0.1 (3)
C18—C9—C10—N1	177.9 (3)	C20—S1—C22—Cl1	−179.3 (2)
